# Therapeutic Potential of Prodrugs Towards Targeted Drug Delivery

**DOI:** 10.2174/1874104501812010111

**Published:** 2018-10-23

**Authors:** Abhinav P. Mishra, Suresh Chandra, Ruchi Tiwari, Ashish Srivastava, Gaurav Tiwari

**Affiliations:** Department of Pharmacy, Pranveer Singh Institute of Technology, Kanpur-Agra-Delhi National Highway (NH-2), Bhauti, Kanpur, Uttar Pradesh, India

**Keywords:** Prodrug, Xenobiotics, Cytotoxic, ADEPT, GDEPT, GPAT, VDEPT, NTR

## Abstract

In designing of Prodrugs, targeting can be achieved in two ways: site-specified drug delivery and site-specific drug bioactivation. Prodrugs can be designed to target specific enzymes or carriers by considering enzyme-substrate specificity or carrier-substrate specificity in order to overcome various undesirable drug properties. There are certain techniques which are used for tumor targeting such as Antibody Directed Enzyme Prodrug Therapy [ADEPT] Gene-Directed Enzyme Prodrug Therapy [GDEPT], Virus Directed Enzyme Prodrug Therapy [VDEPT] and Gene Prodrug Activation Therapy [GPAT]. Our review focuses on the Prodrugs used in site-specific drug delivery system specially on tumor targeting.

## 1 INTRODUCTION

As per definition given by the International Union of Pure and Applied Chemistry (IUPAC), Prodrugs are the chemically modified active drug that has to produce biological and chemical transformation before showing the pharmacological responses [1]. The prodrugs can be thought of a molecule containing nontoxic groups that are required for eliminating the undesirable effect [2]. Furthermore, advanced and sophisticated prodrug design can confer the better pharmacokinetic parameters, prolonged action, increased selectivity, increased membrane permeability, less adverse effects, *etc* [3]. As of now, 5-7% of the drugs that have been approved are the prodrugs. In majority of cases, prodrugs have been found to be a simple chemical derivative that needs one or two enzymatic or chemical transformation for yielding the active drug [4].

Targeted or site-specific drug delivery is one of the basic requirements in controlled drug delivery. In designing of prodrugs, targeting can be achieved by site-directed or site-specific drug bioactivation, such as localized drug delivery for ophthalmic preparation. Prodrugs may be designed for targeting specific enzymes or their carriers by acting on enzyme-substrate specificity or carrier-substrate specificity in order to minimize undesirable drug responses [5]. This type of “targeted-prodrug” requires remarkable knowledge of particular enzymes or carrier systems [6].

## 2 TYPES OF DRUG TARGETING

### 2.1 Tumor Targeting

Selective delivery of anticancer drugs to tumors, without affecting to normal tissues of the body, is one of the major challenges in the treatment of tumor [7]. Synthesis of Prodrugs and their targeting towards specific enzymes based on monoclonal antibodies produces considerable flexibility in experimental designing [8].

### 2.2 Brain Targeting

This is a general and systematic delivery method which can provide sustained release localized effect for a variety of therapeutic agents especially for neuropeptides [9]. With the help of using a sequential approach for drug metabolism, they help to exploit the specific properties of the blood-brain barrier and also provide site-specificity or site-enhanced targeting of the drug substance [10].

### 2.3 Kidney Targeting

**Fig. (1):**
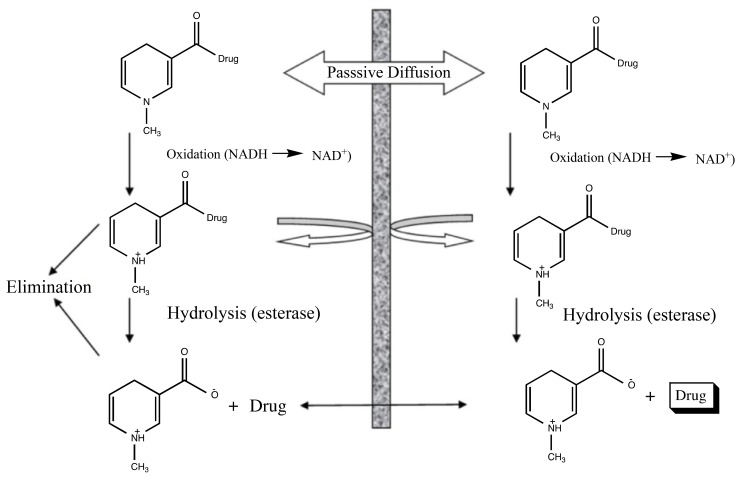
Principle of chemical drug delivery system (CDS).

Renal-specific drug targeting of prodrugs may be an attractive approach in conditions when the drug reaches the kidney cause undesirable extrarenal effects or when renal abnormalities conditions such as in improper GFR, and tubular secretion which may affect the normal renal distribution of a drug to great extent (Fig. **1**) [11].

### 2.4 Colon Targeting

The colon is an only site in our body where both topical, as well as systemic delivery of drugs, can take place. Topical delivery provides local responses in the management of Inflammatory Bowel Disease (IBD). However, treatment can be more effective if the drugs can be made for direct targeting into the colon with minimized systemic side effects. In addition to topical therapy, the colon can also be used as a portal for the entry of drugs into the systemic circulation (Tables **1**, **2**) [12].

## 3 VARIOUS TECHNIQUES OF PRODRUGS EMPLOYED IN TUMOR TARGETING

Nowadays, accurate tumor targeting plays a fundamental role in the therapy of tumors. Precise tumor targeting is required for maximum action with least toxicity. In recent times, several techniques of prodrugs are employed for such purposes, which are as follows:

### 3.1 Hypoxia Selective Prodrug Therapy

**Fig. (2):**
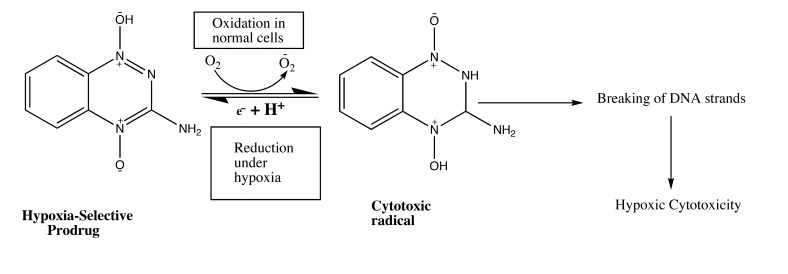
Scheme showing mechanism of action hypoxia selective prodrug (Tirapazamide).

According to the research, in a solid tumor, there is highly irregular blood flow, with the development of oxygen-deficit areas (hypoxia) which is responsible for poor drug delivery thus hypoxic cells may be treated as therapeutic targets for tumor targeting with the help of bioreductive prodrugs. These therapeutic targets for hypoxia-selective drugs depend upon the presence of highly expressed reductase enzymes in tumor cells which reduce bioreductive prodrugs into active cytotoxic radicals under influence of hypoxia (Table **3**) [13, 14]. Under aerobic conditions of normal cells, the radicals oxidized to the nontoxic prodrug with the production of superoxide radical. There are only three types of hypoxia- selective prodrugs that can be used or being developed for their clinical use: Quinine derivatives, Nitroimidazoles, and N-oxides [15]. The N-oxide derivative like Tirapazamide (TPZ) is the first drug to be introduced for hypoxia selective Prodrug in the presence of cytochrome P450, NADPH, oxidoreductase, xanthine oxidase, and aldehyde dehydrogenase. Tirapazamide (TPZ) is biologically reduced to mono deoxygenated toxic products. Under hypoxia, the oxidizing (Fig. **2**) radical leads to the breaking of strands of DNA, which kills the tumor cell [16].

### 3.2 Antibody-Directed Enzyme Prodrug Therapy (ADEPT)

**Fig. (3):**
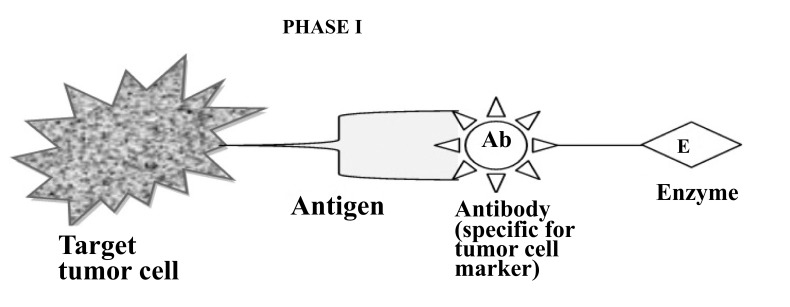
Formation of antibody enzyme complex (Abzyme).

**Fig. (4):**
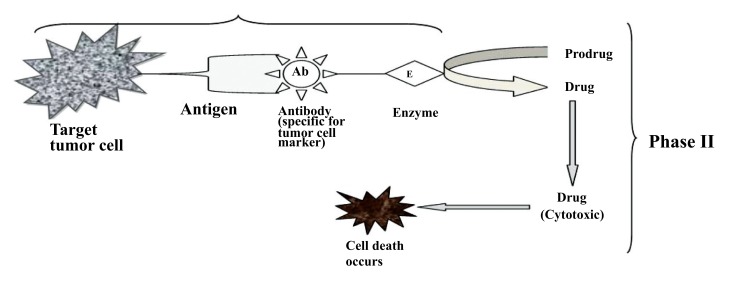
Conversion of prodrug to drug (cytotoxic) and occurrence of death of tumor-affected cell.

To improve the selectivity of anticancer drugs, Antibody-Directed Enzyme Prodrug Therapy (ADEPT) is a therapeutic strategy for targeting tumors. Selectivity for the target In ADEPT is achieved by an antibody (Ab) in an Ab-enzyme complex which binds to the specific antigen situated on the surface of tumor cells. The two-phase antibody targeting system in ADEPT is advantageous over a one-step chemo or radiotherapy [17]. **Phase I-** In this phase, the Ab-enzyme complex is administered that accumulates at the tumor site. For the particular tumor cell, a specific targeted antibody has been used and the enzyme chosen for the conjugate [18] is one that will be used to break the carrier group leave off from the prodrug available in the next phase (Fig. **3**). **Phase II**- after the Ab -enzyme complex has stuck on the tumor cell and the excess conjugate is removed from the blood and normal tissues, then prodrug is administered. The enzyme conjugated with the body at the tumor cell surface bioactivated the conversion of the prodrug to the drug when it reaches the tumor cell (Fig. **4**) [19]. The main advantages of this therapy are to enhance selectivity for targeted cell. A Single enzyme is capable to convert a number of prodrugs molecules. The drug is released at the site of action. The main disadvantages are immunogenicity and rejection of antibody-enzyme conjugate.

### 3.3 Components of ADEPT

Enzymes in ADEPT Specific enzymes are required for ADEPT technique. They should be able to catalyze the conversion of prodrug. From any endogenous enzyme, they should have different catalytic properties. Under any physiological conditions, they should be active and stable. They also prove to be significantly beneficial if they are capable to activate the majority of anticancer prodrugs (Table **4**) [20]. Three different categories of enzymes are used for ADEPT **(i)** enzyme and their homologues originated from other than mammalian species, * e.g.*, β-lactamase (β-L); Penicillin G Amidase (PGA); Carboxypeptidase G2 (CPG2); Cytosine Deaminase (CD) and Penicillin V Amidase (PVA). To minimize the toxicity, these enzymes reduce the risk of activation of the Prodrug by its endogenous enzymes which are present in vascular and normal tissues [21-25]. **(ii)** the enzyme is nonmammalian while their homologues are originated from mammalian species, * e.g.*, β- glucuronidase (β-G) [26]. **(iii)** enzyme and their homologues originated from mammalian species * e.g.*, α- Galactosidase (α-g) and Alkaline Phosphatase (AP) [27]. Antibodies in ADEPT ensure the selectivity of the localized activation of prodrug; the Abs which bind to tumor-specific antigens are a key factor in ADEPT [28]. Ab-conjugates used in ADEPT must have specificity for tumor cells, with high affinity being the main requirement for ADEPT delivery [29]. These Ab conjugates should have minimum binding capacity towards normal tissues. To ensure rapid clearance of the conjugate from body fluids with an addition, the covalent binding of the enzyme must not destroy the ability of the Ab to bind with associated antigen in ADEPT delivery [30]. There are two approaches which oppose Ab-enzyme conjugate to penetrate the tumors. Firstly, the `leaky' vascular bed and interstitium of tumor as compared to that of normal tissue, which is advantageous for the localization of macromolecules [31, 32] and other one the random distribution which leads to decrease the uptake of macromolecules [33, 34]. This limitation can be overcome by using Ab fragments, * e.g.*, F (ab′) 2, F (ab′) and scFv, which increase rate of transport into the interstitial region of tumor. These fragments have better penetration ability than intact Ab and also show more rapid clearance as demonstrated in animals and in patients [35].

### 3.4 Use of Prodrugs in ADEPT

The major problem for tumor therapy is poor vascularisation of tumors cells. To produce more effective treatment towards the delivery and penetration of molecules across the physiological barriers of the tumor are an extremely important parameter in this approach. There are two factors which govern the uptake of a compound into the tumor, one is the extraction coefficient by the tumor and second is the blood flow in the vascular portion in the tumor. Lipophilicity and properties of the physiological barrier play an important role for extraction of Prodrug by the tumor through the blood flow. The factors through which a prodrug development can meet optimum availability through the blood flow are a possibility for leak back of the drug from the tumor and the pharmacokinetic properties of the prodrug [36]. In designing of prodrug through ADEPT should be less cytotoxic than their corresponding active component (Table **5**).

The prodrug designing through this technique should be chemically stable under targeted physiological conditions and must have good pharmacodynamic and pharmacokinetic responses as well as the prodrugs substrates must have properties for activation when it attached on enzyme under targeted physiological environment. ADEPT prodrugs are derived from well-known anticancer agents or their analogues as model molecules for the designing of a less cytotoxic prodrug to release a highly toxic drug requires adequate knowledge of their Structure-Activity Relationships (SAR) towards its cytotoxic action. Well, known pharmacokinetic parameters of the drugs give an additional advantage for this delivery. Since a change in one physicochemical property of the prodrug, structure affects a variety of the properties of that prodrug (* e.g.*, biodistribution, reactivity, and pharmacokinetics) in addition to the enzyme kinetics [37].

### 3.5 Gene-Directed Enzyme Prodrug Therapy (GDEPT)

Gene-Directed Enzyme Prodrug Therapy (GDEPT) is a technique which physically delivers a gene for a foreign enzyme to tumor cells where a systemically administered nontoxic prodrug can be activated when enzymes are expressed. This is also known as suicidal gene therapeutic phenomenon [38]. GDEPT can also be used to improve the selectivity of currently used medicaments *via* CYP activation. Prodrug activation with the help of CYP activation system is one of the good examples of GEDPT delivery (Table **6**).

Members of the CYP enzyme superfamily convert the anticancer agents, cyclophosphamide and ifosfamide are acted *via* alkylating agents which cause cell death [39]. Generally, the liver has overexpressed CYP as compared to tumor cells, which leads to action for intrinsic drug resistance. Recently, CPA/MTX-α-peptide system was developed to improve its mechanism. CPA is a zymogen that becomes catalytically active after detachment of its propeptide portion in the presence of trypsin [40]. Thus, Activated CPA converts MTX-α-peptide prodrug into active MTX (Methotrexate) that inhibits Dihydrofolate Reductase (DHFR) and eventually causes cell death [41]. As trypsin is most abundantly present in the small intestine but it is found absent in tumors thus, the prodrug activation by CPA is limited to the intestine, causing local toxicity and low drug concentration in tumors. To activate the prodrug at tumor site in a trypsin-independent manner, a battery of CPA mutants is developed in which the trypsin cleavage sites cause mutation at the site of mammalian propeptidases recognition [42].

**Fig. (5):**
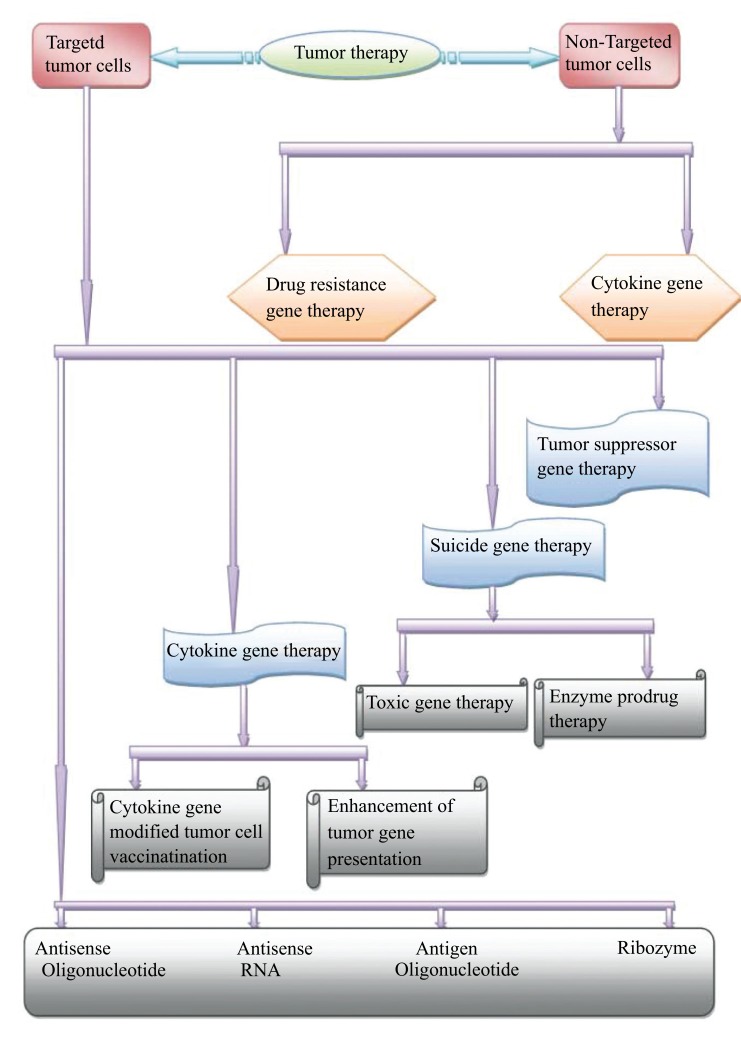
Flowchart showing various modes of targeting used in cancer or tumor therapy.

GDEPT technique could be used to treat any solid tumor that is accessible either directly or *via* local perfusion. The technology could also be beneficial in treatment strategies for other diseases, such as graft versus host disease. Gene-Directed Enzyme Prodrug Therapy (GDEPT) is a two-step treatment approach where the gene for a non-endogenous enzyme is directed to target tissues. The expression of enzyme at tumor site are able to activate a simultaneously administered prodrug. It is a new and promising treatment for current cancer chemotherapy (Fig. **5**) [43].

**Fig. (6):**
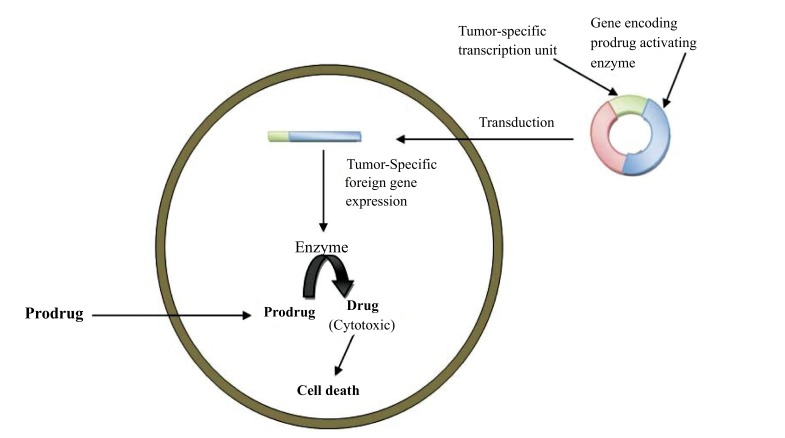
Mechanism of GDEPT technique in treatment of tumors.

The requirement in GDEPT therapy is a non-endogenous enzyme produced by a gene (expression of enzyme at low level) which is responsible for the activation of a prodrug and injection of the prodrug. To achieve success in GDEPT, two factors must be taken into consideration such as, firstly, the targeted gene should only be expressed in the tumor cells, and secondly, overexpression in the tumor cells should be as high as possible (Fig. **6**) [44].

### 3.6 Genetic Prodrug Activation Therapy (GPAT)

Genetic Prodrug Activation Therapy (GPAT) technique is an improved form of Gene-Directed Enzyme Prodrug Therapy (GDEPT) technique. This strategy works upon intracellular conversion of a relatively non-toxic prodrug into a toxic drug by a xenobiotic origin enzyme and has been referred to as genetic prodrug activation therapy (GPAT) [45]. Genetic Prodrug Activation Therapy (GPAT) is a newer approach that can destroy tumor cells by inserting 'suicide' genes into tumor cells. Transcriptional differences between normal and tumor cells employ to drive the selective expression of a metabolic “suicide gene” that is able to convert a nontoxic prodrug into its toxic metabolite (drug) in this therapy [46]. The most common description of GPAT strategy is seen in the Herpes Simplex Virus thymidine kinase (HSVtk) enzyme ganciclovir (GCV) prodrug system [1]. HSVtk may act through phosphorylation of GCV that results in product incorporating into DNA during cell division which ultimately results in the death of tumor cell [47].

### 3.7 Virus-Directed Enzyme Prodrug Therapy (VDEPT)

**Fig. (7):**
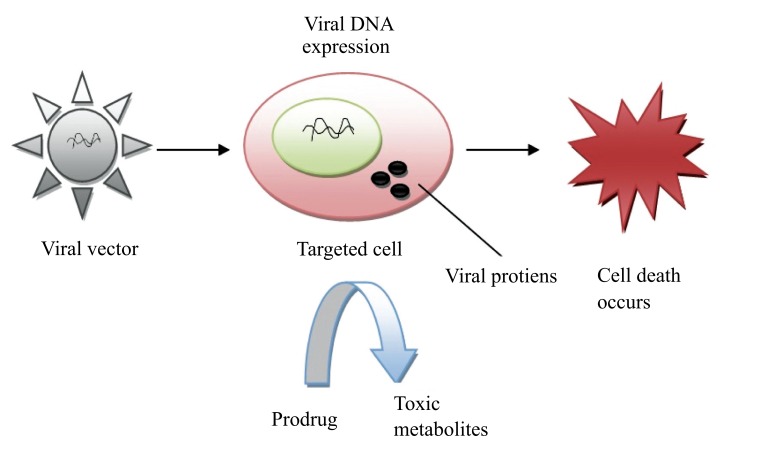
Mechanism of VDEPT technique in cancer treatment.

Virus-Directed Enzyme Prodrug Therapy (VDEPT) is an emerging strategy for the treatment of tumors [48]. This technique uses viral vectors for the introduction of a transgene that is more specifically referred to as VDEPT technique. In this approach, a viral vector encoded by an enzyme which can convert inactive prodrug into a cytotoxic metabolite that infects the tumor cell. Upon prodrug administration, an enzyme coded by a viral vector in the tumor cell leads to produce cytotoxic metabolite which results in direct tumor cell death (Fig. **7**).

A study revealed that when the *E. coli* NTR (nitroimidazole reductase) gene was introduced into colorectal and pancreatic cancer cell lines by retroviral delivery, it was observed that NTR-expressing clones of both cell lines were more susceptible to cytotoxic effects mediated by the prodrug CB1954 [30], which revealed that NTR and CB1954 [5-[aziridin-1-yl]-2, 4-dinitrobenzamide] can be used as an attractive combination for treatment of tumors that employs VDEPT technique. NTR (nitroimidazole reductase) has also acted on tumor cells by a replication-defective adenovirus vector containing an NTR expression. It also suggested the high sensitivity of NTR-expressing cells to CB1954. Additionally, cisplatin-resistant cells with NTR expression were also found to be susceptible to CB1954, suggesting that this system may also be useful for tumor treatment in patients with cisplatin-resistant tumors (Table **7**) [49].

## 4 RESULT

Nowadays, the treatment for tumor is one of the biggest challenges in the medical field as the drugs used for this purpose produce a wide range of side effects due to lack of selectivity, the solution to this problem can be achieved by the development of different strategies of prodrug targeting or site specificity (GDEPT, ADEPT, VDEPT *etc*.). To enhance aqueous solubility, the prodrug approach has been used as a successful tool. To avoid discarding promising active prototypes or drugs with therapeutic uses, the prodrug approach can make it possible to achieve this goal. The rational selection of pro-moiety and the type of linkage may determine the prodrug selectivity, toxicity, and ideal bioconversion profile towards its site specificity. All the prodrug- targeting techniques are practically advantageous for optimizing the treatment of tumor cells. GDEPT and VDEPT are somewhat advantageous over ADEPT as most of the enzymes need cofactor[s] which are present only inside the cells. Still, the choice of GDEPT, VDEPT and ADEPT for the treatment of tumors should always depend on the clinical scenario.

## CONCLUSION

Furthermore, the prodrug approach could be viewed as an alternative in the early phases of drug discovery. This strategy may be useful to enhance pharmacokinetic properties (ADME), as well as poor aqueous solubility, which clears a critical step in pre-clinical phase drug development.

## Figures and Tables

**Table 1:** Colon targeting sites, diseases and drugs for their therapy.

**Type of Action**	**Disease Conditions**	**Drug for Therapy**
Topical Action	Inflammatory Bowel Diseases, Irritable bowel disease and Crohn’s disease. Chronic Pancreatitis.	Hydrocortisone, Budenoside, Prednisolone, Sulphasalazine, Olsalazine, Mesalazine, Balsalazide.
Local Action	Pancreatactomy and cystic fibrosis, Colorectal cancer	Digestive enzyme supplements, 5-Flourouracil
Systemic Action	To prevent gastric irritation To prevent first pass metabolism of orally ingested drugs Oral delivery of peptides Oral delivery of vaccines	NSAIDS Steroids Insulin Typhoid

**Table 2:** Examples of prodrugs used for site specific drug targeting.

**** **S. No.**	**Types of Targeting**	**Examples**	**Structure**
**1.**	**Tumor Targeting**	**Doxorubicin**	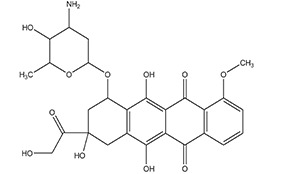
**2.**	**Brain Targeting**	**Zidovudine**	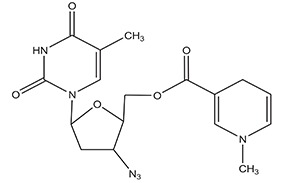
**3.**	**Kidney Targeting**	**γ glutamyl levodopa**	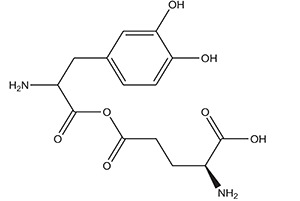
**4.**	**Colon Targeting**	**Sulphasalazine**	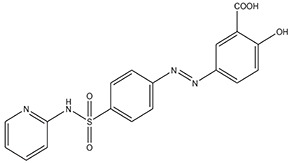

**Table 3:** Other examples of hypoxia selective prodrugs used for tumor targeting.

**S. No.**	**Category**	**Examples**		**Structure (Prodrug)**
		**Prodrug**	**Drug**	
1.	Quinone derivatives	Porfiromycin	Mitomycin C	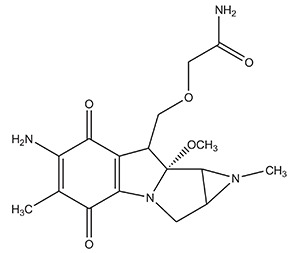
2.	Nitroimidazole derivatives	RB6145 (Bromoethylaminonitroimidazoylpropanolol)	RSU1069 (Aziridinylnitroimidazoylpropanolol)	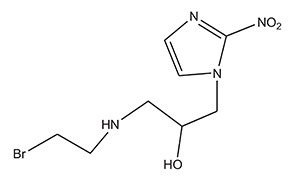

**Table 4:** Some characteristics of enzymes used for ADEPT systems.

**Enzyme**	**Source**	**Reaction Specificity**
AP	Calf intestine	Hydrolysis of phosphate groups from aliphatic and aromatic substrates
CPA	Bovine pancreas	Cleavage of α-glutamyl-peptides, * e.g.* Methotrexate release from methotrexate-α-peptides.
CPG2	*Pseudomonas sps.*	Cleavage of amidic, oxycarbonyl and carbamic bonds located between an l-glutamyl moiety and an aromatic nucleus.
CD	Baker’s yeast	Catalyses the deamination of cytosine to uracil
α-g	Mammalian	Hydrolysis of α-galactosyl-linked residues; used with a self-immolative linker
β-g	*E. coli*	Hydrolysis of β-galactosyl-linked residues
β-G	*E. coli* (type X-A); *E. Coli* K12	Hydrolysis of glucuronides linked to various substrates
β-glu	Sweet almonds	Hydrolysis of β-glucose-linked residues
β-L	*Enterobactercloace* strain 256A; *B. cereus*	Cleavage of the 4-membered lactam of cephalosporin: effects elimination of substituents appended to 3′-position of cephalosporin substrates
NR	*E. coli*	Reduction of nitro groups in some aromatic systems
PGA	*E. coli*	Cleavage of the phenyloxyacetamide groups linked
PVA	*Fusariumoxysporum*	Cleavage of the phenyloxyacetamide groups linked to various substrates

**Table 5:** Some examples of prodrugs used for ADEPT system.

**S.No.**	**Category**	**Examples**	**Structure**
1.	Alkylating Agents	4[(2-chloroethyl)(2-mesyloxyethyl)amino]benzoyl-l-glutamate	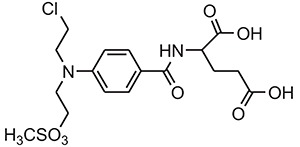
2.	Enediynes	Esperamycin	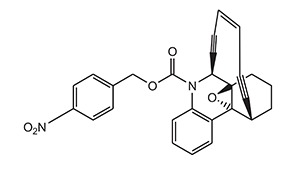
3.	Antimetabolites	5-fluorouracil	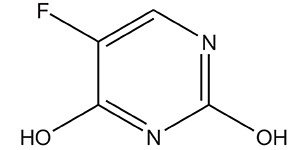
4.	Natural Anticancer Prodrugs	Anthracyclin antibiotics (doxorubicin-phosphate)	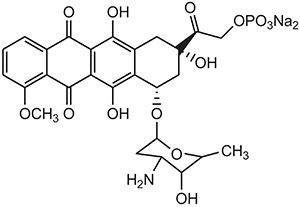

**Table 6:** Some examples of prodrugs employed in GDEPT technique.

**S.No.**	**Category**	**Examples**	**Structure**
1.	Alkylating agents	Cyclophosphamide	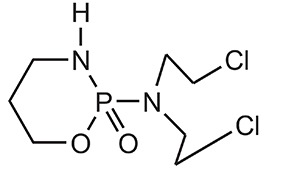
		Isophosphamide	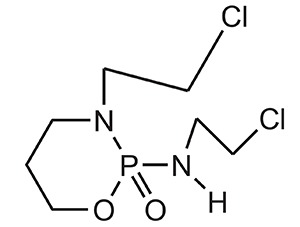
2.	Anthracyclines	HMR 1826 (Doxorubicin)	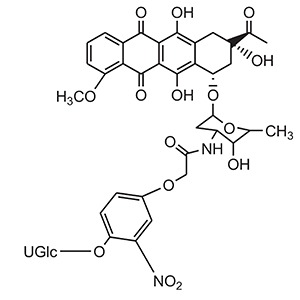

**Table 7:** Viral vectors and Enzymes used for VDEPT system.

**S.No.**	**Viral vectors**	**Enzymes**	**Examples**
1.	Adenovirus	Thymidine kinase	Ganciclovir
		Human carboxylesterase	Iirinotecan
		Nitroreductase	CB 1954
2.	Retrovirus	Nitroreductase	CB 1954
		Cytosine deaminase	5-FC
		Human CYP and P450 reductase	Cyclophosphamide
3.	EBV	Nitroreductase	CB 1954
